# A Rare Case of Intravascular Large B-cell Lymphoma Presenting With Bilateral Ophthalmoplegia, Along With a Literature Review

**DOI:** 10.7759/cureus.25920

**Published:** 2022-06-14

**Authors:** James Allen, Anam Shaikh, Karrah Laurent-Ariot, Matthew Merola

**Affiliations:** 1 Internal Medicine, St. John's Episcopal Hospital, New York, USA; 2 Psychiatry, St. John's Episcopal Hospital, New York, USA; 3 Neurology, State University of New York Downstate Medical Center, Brooklyn, USA; 4 Neurology, State University of New York Downstate Health Sciences University, Brooklyn, USA

**Keywords:** secondary headache, case report, intravascular large b cell lymphoma, ptosis, bilateral ophthalmoplegia

## Abstract

Intravascular large B-cell lymphoma (IVLBCL) is a subtype of extranodal lymphoma that characteristically contains malignant lymphocytes within blood vessels. The clinical presentation of IVLBCL has high variability. In our case, the patient’s initial presentation involved bilateral ptosis, restricted extraocular movements, periorbital pain, and bitemporal headache. The patient denied the classic “B symptoms” such as fever, night sweats, or weight loss. The patient also denied a family history of malignancy. Initial imaging studies were unremarkable, making diagnosis particularly challenging. Ultimately, functional endoscopic sinus surgery was performed. Pathological examination of the intraoperative specimen revealed a CD5+ large B-cell lymphoma within the vessels involving the left ethmoid sinus, respiratory mucosa, and nasal septum. The patient underwent steroid therapy prior to diagnosis, which led to rapid improvement in headache and mild improvement in extraocular function and ptosis. Following diagnosis, the patient underwent chemotherapy with supportive medications. Our case report may be considered a reference for cases presenting with extensive bilateral extraocular muscle deficits and levator palpebrae dysfunction in the absence of notable initial imaging findings, “B symptoms,” or positive family history. The teaching point from this case is to demonstrate the difficulty of diagnosis and our train of thought in investigating an abnormal presentation with no clearly identifiable etiology following initial diagnostic workup and treatment.

## Introduction

Intravascular large B-cell lymphoma (IVLBCL) is a rare subtype of extra-nodal lymphoma characterized by malignant lymphocytes only in the lumina of medium and small-sized blood vessels. The clinical presentation varies significantly, with no clinically useful biomarkers or consistent imaging findings, making clinical suspicion difficult. Clinical manifestations include signs and symptoms related to the involved organs. The potential organs involved include the bone marrow, spleen, liver, central nervous system (CNS), skin, and blood [1]. IVLBCL is often considered disseminated at onset due to these difficulties. Despite the heterogeneity in presentation, the skin and CNS are the most frequently affected, with over 60% of the known cases being complicated by neurological manifestations [1]. Herein, we report the case of a patient with right supraorbital pain, bilateral ptosis, fixed globe, and blurry vision, demonstrating the difficulty in the diagnosis of IVLBCL due to several possible differential diagnoses.

## Case presentation

A 73-year-old man with a history of hypertension presented with bilateral extraocular movement restriction and drooping of the eyelids (left > right) for one month. Drooping started in the right eye one month prior to presentation and progressed to involve the left eye. The symptoms were intermittent during the day and worsened during nighttime. The patient also complained of a bitemporal and periorbital headache for three days, which was improved with Acetaminophen. The patient had no other complaints. The patient was admitted to the Neurology department for further workup.

**Figure 1:**
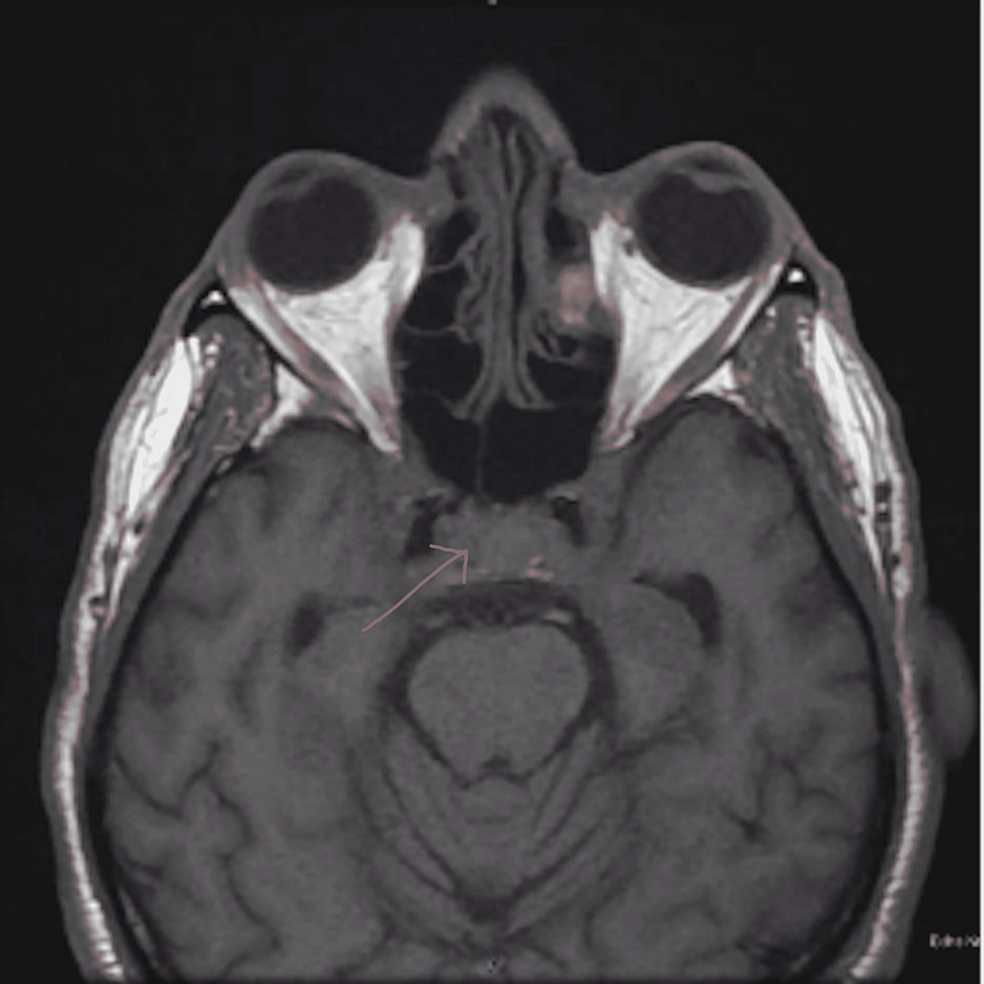
Pre-contrast T1 MRI Brain Demonstrating Diffuse Abnormal Thickening and Enhancement of the Cavernous Sinus

**Figure 2:**
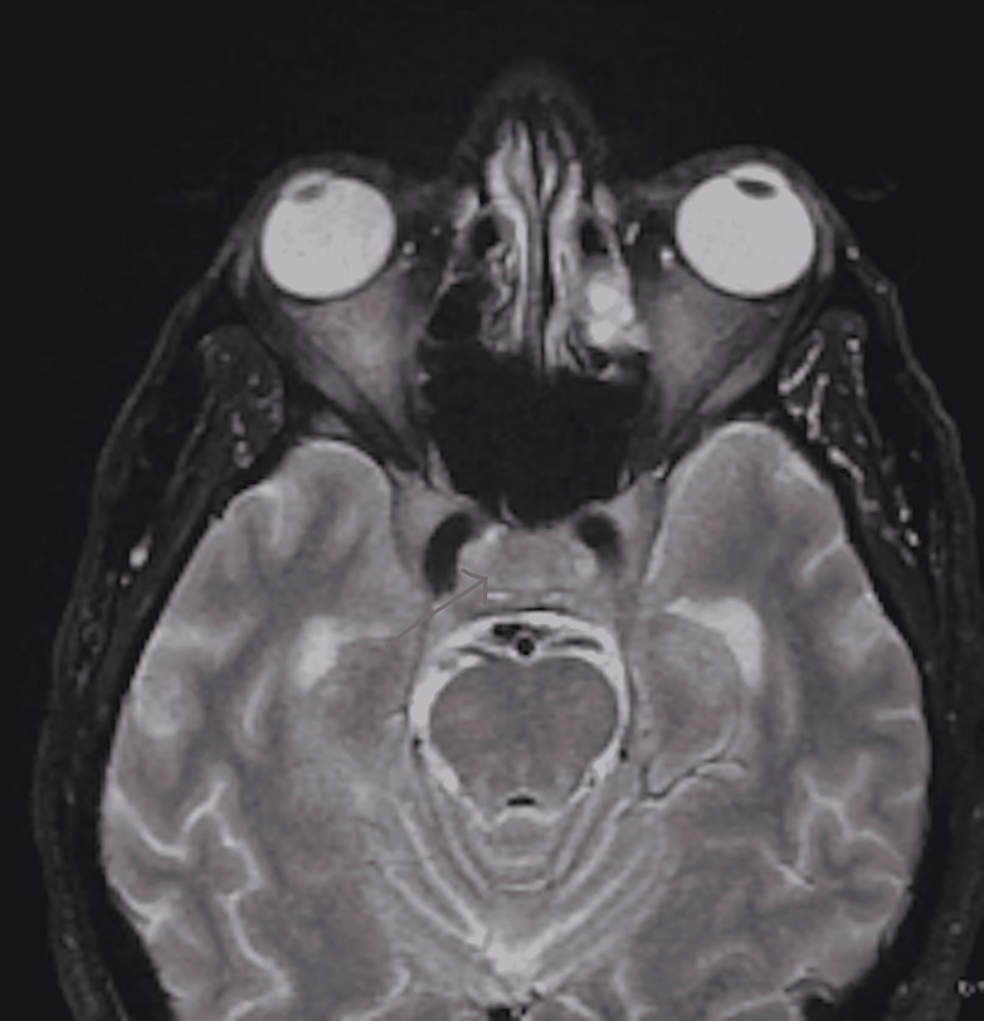
Pre-contrast T2 MRI Brain Demonstrating Diffuse Abnormal Thickening and Enhancement of the Cavernous Sinus

**Figure 3:**
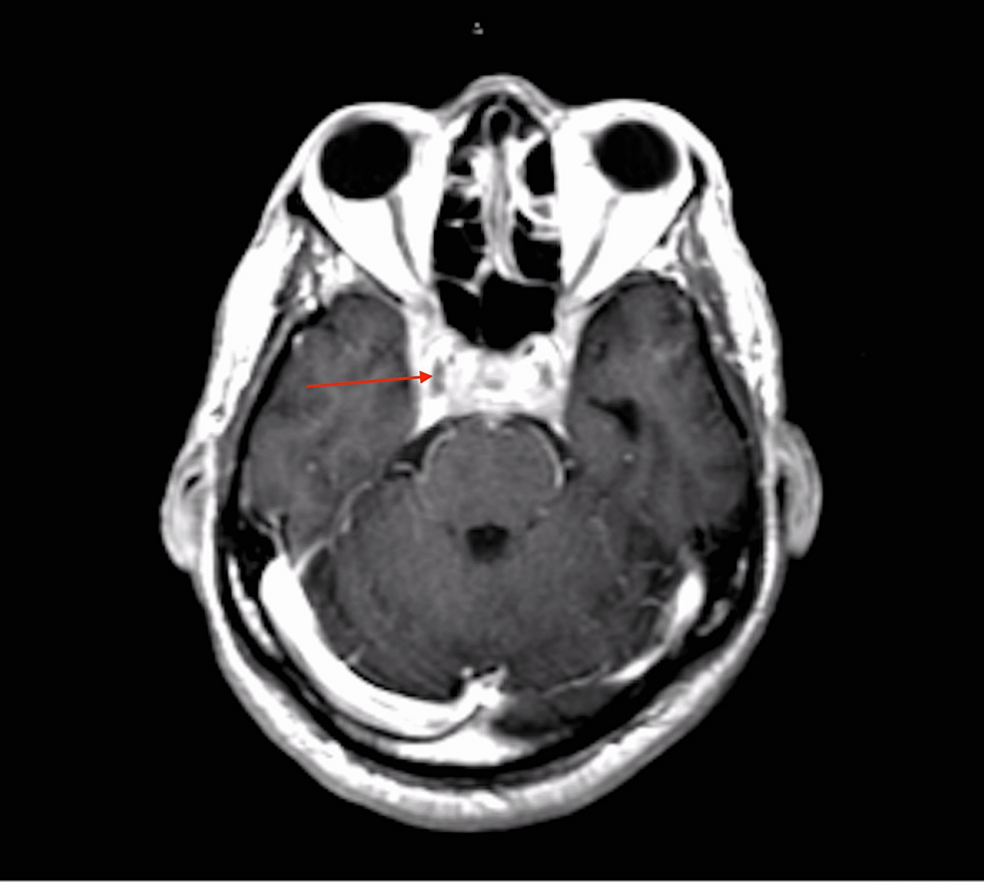
Post-contrast T1 MRI Brain Demonstrating Diffuse Abnormal Thickening and Enhancement of the Orbital Apices, Superior Orbital Fissures, Cavernous Sinuses, and Sella

**Figure 4:**
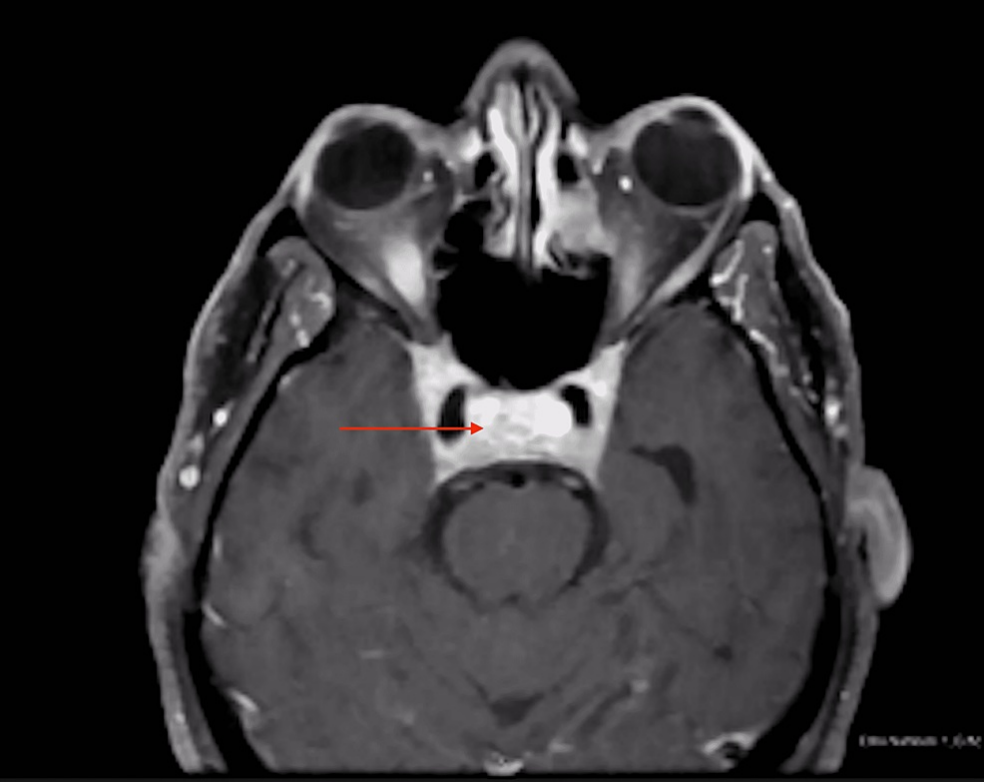
Post-contrast T1 Fat-Sat MRI Brain Demonstrating Diffuse Abnormal Thickening and Enhancement of the Orbital Apices, Superior Orbital Fissures, Cavernous sinuses, and Sella

The patient's vital signs were stable, except for a blood pressure of 155/99 mmHg. Physical examination revealed severe bilateral extraocular movement (EOM) restriction, ptosis (left > right), and bilateral constricted visual fields (tunnel vision). Non-contrast head CT performed two weeks prior showed complete opacification of the left frontal sinus, indicating acute sinusitis. Due to the multitude of neurological symptoms, brain magnetic resonance (MR) imaging, angiography, and venography were performed with and without contrast. The MR images showed diffuse abnormal thickening and enhancement of the orbital apices, superior orbital fissures, cavernous sinuses, sella, and mild left frontal sinusitis (Figures 1-4). The neuroradiologist initially suspected Tolosa-Hunt syndrome. The patient was administered three doses of dexamethasone 10 mg q8hrs and intravenous ampicillin-sulbactam to treat the sinusitis. Subsequently, steroid therapy was changed to intravenous methylprednisolone 80 mg daily. As Tolosa-Hunt syndrome is a rare disease and a diagnosis based on exclusion, further tests were performed to identify other possible conditions.

Sinus CT with intravenous contrast revealed findings similar to those of the brain MRI concerning of Tolosa-Hunt syndrome, with additional findings of focal paranasal sinus disease (Figures 1-4). Chest CT without contrast was performed to rule out sarcoidosis, which revealed bilateral prominent axillary lymph nodes. One of the initial differential diagnoses was myasthenia gravis, for which a repetitive stimulation test was performed that showed negative results. Furthermore, investigations for inflammatory causes revealed a mildly elevated erythrocyte sedimentation rate (63 mm/hr). Antinuclear antibody screening revealed positive cytoplasmic, reticular pattern, and coarse granular filamentous staining extending throughout the cytoplasm. Other laboratory tests include complete blood count, complete metabolic panel, thyroid function test, human immunodeficiency virus (HIV), rapid plasma reagin (RPR), urinalysis, urine drug screening, coronavirus disease 2019 (COVID-19) polymerase chain reaction (PCR), C-reactive protein (CRP), anti-double-stranded DNA (anti-dsDNA) antibodies, anti-acetylcholine receptor antibodies, anti-muscle-specific kinase (anti-MuSK) antibodies, anti-smooth muscle antibodies, voltage-gated calcium channel antibodies, rheumatoid factor, and complements 3 and 4 revealed negative results. The results of cerebrospinal fluid (CSF) analysis, including protein, glucose, venereal disease research laboratory (VDRL), and DNA PCR for Lyme disease, were within normal limits.

The patient's headache and right extraocular movement (EOM) abnormalities started to improve after two days of high-dose steroid therapy. However, since the ptosis in the left eyelid failed to improve, functional endoscopic sinus surgery of the ethmoid sinus was performed, as the ENT specialist suspected that the sinusitis might be related. Pathological examination revealed an intravascular diffuse large B-cell lymphoma that was CD5+ within the vessels involving the left ethmoid sinus, respiratory mucosa, and nasal septum. A bone marrow biopsy was performed by the oncology team, which was negative for evidence of lymphoma. CT of the abdomen/pelvis was performed to detect metastasis, which revealed an 11 mm sclerotic focus in the left acetabulum and heterogeneous mixed lytic sclerotic changes in the T8 vertebral body. Protein electrophoresis for multiple myeloma showed negative results.

As the patient’s headache and ptosis improved, the patient was switched to oral prednisone 80 mg, which was subsequently tapered. The patient was discharged with recommendations to start chemotherapy in the outpatient setting.

## Discussion

IVLBCL is a subtype of large-cell lymphomas. The characteristic findings of large cell lymphomas are the proliferation of the lymphoma cells within the lumen of blood vessels with an aggressive clinical course. IVLBCL is rare; therefore, the majority of the data are case reports and expert panel opinions [1]. Although the true incidence of IVLBCL is unknown, some studies have reported that the incidence is less than one person per million [2-3]. The average age at the time of diagnosis is in the sixth and seventh decades of their lives, and men and women are affected equally [2,4-5]. A genetic component may be observed in IVLBCL [6-8]. IVLBCL can present with a multitude of symptoms depending on the vessels affected. Although the CNS and skin are more commonly affected, axonal polyneuropathy, pancytopenia, and multiple strokes may also be seen [2,9-13]. Furthermore, CNS and skin involvement are more common in Western countries than in Asian countries [2]. However, Asian patients frequently present with end-organ, such as bone marrow (75%), spleen (67%), and liver (55%), involvement [14].

The diagnosis of IVLBCL is often challenging due to vague symptoms, with several other conditions mimicking the disease. Traditionally, such cases have been diagnosed during an autopsy [5,12]. To date, the diagnosis of IVLBCL relies on a combination of clinical features (multiple admissions and progressive symptoms) and pathological findings on biopsy [3]. The consensus on biopsy strategy is poor. Some experts recommend deep skin biopsies or at least three specimens of random skin biopsies. As in the present case with brain involvement, other experts recommend obtaining a skin biopsy followed by a brain biopsy [15]. Considering that many organs might be affected, biopsies can be obtained from other tissues if the skin biopsy is non-diagnostic [1,13]. Other findings that can aid in diagnosis include elevated lactate dehydrogenase, β2-microglobulin levels, and anemia.

Considering the limited data available, treatment of IVLBCL is similar to that of diffuse large B-cell lymphoma. The treatment includes a combination of cyclophosphamide, doxorubicin, vincristine, and prednisone with recombinant anti-CD20 antibody rituximab [1-2,16-20]. One of the largest retrospective studies conducted on 106 patients were treated with either chemotherapy alone (57%) or chemotherapy plus rituximab (49%) [17]. Response to treatment may vary depending on the organs involved; however, studies have reported generally favorable responses, with some patients showing complete remission [2-3].

## Conclusions

Intravascular large B-cell lymphoma is a difficult disease to recognize, requiring a high degree of clinical suspicion. We presented a case that highlighted the variable nature of IVLBCL with an atypical set of symptoms. An extensive workup was performed, ruling out the mimickers and ultimately obtaining biopsy samples demonstrating a CD5+ large B-cell lymphoma within the vessels, involving the left ethmoid sinus, respiratory mucosa, and nasal septum. The patient was treated with steroids and chemotherapy with significant improvement. In conclusion, we have presented a case of IVLBCL with bilateral ophthalmoplegia to help demonstrate our critical thinking process and the difficulty in the diagnosis of IVLBCL.
